# Rhegmatogenous and tractional retinal detachment in eyes with retinoblastoma: a retrospective surgical case series from a tertiary eye center

**DOI:** 10.1186/s40942-026-00858-7

**Published:** 2026-05-29

**Authors:** Hend AlSafran, Marcos J. Rubio Caso, Gorka Sesma, Adhwa Alsadoon, Abdullah M. Khan, Saleh AlMesfer

**Affiliations:** 1 Vitreoretinal Division, King Khaled Eye Specialist Hospital & Research Center, Riyadh, Saudi Arabia; 2 Pediatric Ophthalmology & Strabismus Division, King Khaled Eye Specialist Hospital & Research Center, West Building, 2nd Floor, Riyadh, 12329 Saudi Arabia; 3 AlDukhan Eye Center, Al-Amiri Hospital, Ministry of Health, Kuwait City, Kuwait

**Keywords:** Retinoblastoma, Rhegmatogenous retinal detachment, Tractional retinal detachment, Vitreoretinal surgery, Surgical outcomes

## Abstract

**Purpose:**

To evaluate the clinical characteristics, management strategies, and surgical outcomes of tractional and/or rhegmatogenous retinal detachment (TRD/RRD) in eyes with retinoblastoma (RB).

**Methods:**

A retrospective surgical case series of patients with treated RB at a tertiary referral center (June 2014–April 2025) who developed non-exudative retinal detachment (RD) was conducted. The data included demographics, tumor activity, RB treatment modalities received, RD features, surgical approach, complications, anatomical reattachment, and final visual acuity.

**Results:**

Seven eyes of seven patients (0.9%) developed TRD or RRD. Four eyes had RRD, two had combined RRD/TRD, and one had TRD. Early RRD during active RB was treated with nondrainage scleral buckling (*n* = 2), whereas late detachments after complete tumor regression underwent pars plana vitrectomy with silicone oil tamponade (*n* = 4). Anatomical reattachment at one year was achieved in 83.3% of the operated eyes (five of six eyes undergoing surgery), with no cases of tumor seeding or reactivation observed. One eye was enucleated due to tumor progression. Visual acuity improved to baseline or better in 71.4% of the eyes.

**Conclusions:**

In this small series, early RRD during active RB was managed with nonsurgical scleral buckling without evidence of tumor dissemination, and late complex RRD after confirmed tumor quiescence was treated with pars plana vitrectomy and silicone oil tamponade with favorable anatomical outcomes. No tumor seeding or reactivation was observed in any case. However, these findings should be interpreted with caution, given the small sample size, and do not allow definitive conclusions regarding oncologic safety.

**Supplementary Information:**

The online version contains supplementary material available at 10.1186/s40942-026-00858-7.

## Introduction

Retinoblastoma (RB) is the most common primary intraocular malignancy in children. Advances in systemic and focal therapies have increased the survival rate to > 95% and have improved global salvage [1, 2]. However, vitreoretinal complications, such as rhegmatogenous retinal detachment (RRD) and tractional retinal detachment (TRD), continue to pose a significant threat to vision in treated eyes, affecting approximately 6–7% of cases [3].

Previous studies have reported a prevalence of RRD following RB treatment ranging from 3% to 11%, typically associated with atrophic breaks occurring in up to 67% of cases at the sites of cryotherapy or laser scars, and delayed changes in the vitreoretinal interface [4, 5]. Additional risk factors include external beam radiation and systemic chemotherapy, which may exacerbate retinal thinning and vitreous traction and impair wound healing processes that may normally protect the retina from detachment [6, 7]. RRD typically presents months to years after RB treatment, often following focal therapy, and may involve less than two quadrants in most cases. Delayed onset, the presence of atrophic retinal holes, and slower progression all help distinguish RRD from exudative detachments, which are more acute and tumor-related [4].

These detachments are surgically challenging because of the risk of tumor dissemination and complex pathologies [6, 7]. In eyes with clinically active RB, studies have reported favorable outcomes using undrainage age scleral buckling, with success rates of 80–100% and no cases of orbital or metastatic spread during follow-up [8]. Vitrectomy has been less commonly employed and is typically reserved for combined tractional components or delayed scenarios; intraocular surgery is preferably delayed in eyes with RB for two years after tumor quiescence [1]. TRD alone is uncommonly seen in eyes with RB and usually presents years after tumor treatment and inactivity; vitrectomy to remove these tractional membranes has been successfully performed in such cases [3].

Despite the extensive literature on Western populations, no published series has described the characteristics and outcomes of TRD/RRD in RB in the Gulf region. At our tertiary center, we commonly observe delayed retinal detachment (RD) presentation, providing a unique clinical spectrum that is not well represented in existing reports.

This case series described the clinical features, surgical management, and anatomical and functional outcomes of TRD/RRD in eyes with RB and assessed the safety of scleral buckling and pars plana vitrectomy (PPV) in these uncommon and complex cases.

## Methods

### Study design and setting

This retrospective observational case series was conducted at the King Khaled Eye Specialist Hospital and Research Center (KKESH&RC) in Riyadh, Saudi Arabia, which serves as the national tertiary referral center for ophthalmic oncology in the Kingdom. A systematic review of electronic medical records identified 766 consecutive patients diagnosed with RB and managed at our institution between June 2014 and April 2025. From this institutional cohort, all cases in which non-exudative RD developed and subsequently underwent surgical repair were selected for inclusion. The number 766 patients is provided solely to contextualize the rarity of this complication and does not serve as a formal at-risk denominator. This study was designed as a descriptive case series focused on the clinical characterization and surgical outcomes of RD complicating RB and was not intended to estimate the incidence or prevalence in globe-salvaged eyes, which would require a prospective cohort design with defined surveillance criteria.

### Eligibility criteria

Patients who subsequently developed RRD, TRD, or combined tractional and rhegmatogenous retinal detachment (CTRRD) were included in this study. Eyes with isolated exudative RD were excluded from the study. In patients with bilateral RB, each eye was analyzed as an independent unit. In the present series, no patient developed RD in both eyes.

### Data collection

Demographic data included date of birth, sex, and date of RB diagnosis. Tumor characteristics collected for each affected eye included laterality, International Classification of RB (ICRB) group, and treatment modalities received [9].

The RD-related variables included the date of diagnosis, affected eye, type of detachment, extent of detachment (in clock-hours), quadrants involved, presence and grade of proliferative vitreoretinopathy (PVR) according to the updated Retina Society Classification, and number, type, and location of causative retinal breaks in eyes with RRD or CTRRD [10]. Fundus images were acquired using an Optos ultra-widefield system (Nikon Corporation, California, USA), and OCT imaging was performed using a Spectralis OCT platform (Heidelberg Engineering, Heidelberg, Germany).

The interval from the last documented evidence of active RB (defined as the date of the last EUA or clinical assessment at which tumor activity was confirmed) to the date of RD diagnosis and from RD diagnosis to the date of RD surgery were recorded for each eye. The sum of these two intervals is reported as the total quiescence-to-surgery interval.

Surgical data included the RD treatment modality, number of surgical procedures, surgical technique (unimanual or bimanual), type of intraocular tamponade when PPV was performed, and intra-and postoperative complications.

Best-corrected visual acuity (BCVA) was recorded at the last visit prior to RD, at the time of RD diagnosis, and at 1, 3, 6, and 12 months thereafter. Visual acuity measurements were converted to the logarithm of the minimum angle of resolution (logMAR) values for analysis, when applicable.

### Surgical technique

All surgical procedures were performed under general anesthesia. PPV was performed using a 23-gauge three-port system with a non-contact wide-angle viewing system. Triamcinolone acetonide (4 mg/0.1 mL) was used to stain the posterior cortical vitreous and facilitate the identification and removal of the posterior hyaloid. After core vitrectomy and posterior vitreous detachment induction, the vitreous base was meticulously shaved under scleral depression to minimize residual peripheral traction. Scleral depression was performed by the assistant surgeon throughout the peripheral shaving. Membrane peeling was performed using end-gripping forceps with the assistance of Trypan blue staining of epiretinal membranes, according to the intraoperative findings.

Tamponade selection was individualized: silicone oil (1,000 cSt) was preferred for eyes with inferior breaks, significant PVR, or when postoperative positioning compliance was uncertain. Heavy silicone oil (Densiron-68^®^ or equivalent) was used in cases with inferior breaks and extensive inferior PVR.

Scleral buckling (SB) was performed as an adjunct or primary procedure in two cases. In Case 6, a segmental radial sponge buckle was placed over the causative break after cryotherapy application to the break margins, using a nondrainage technique with intraoperative intraocular pressure (IOP) monitoring. In Case 7, a segmental/circumferential buckle was placed without cryotherapy. In both SB cases, the decision to avoid drainage was guided by the risk of choroidal hemorrhage and the plan for concurrent fluid–air exchange.

In Case 5, which presented with secondary CTRRD to extensive fibrovascular proliferation, relaxing retinotomy with panretinal photocoagulation (PRP) was required to achieve retinal flattening, followed by silicone oil tamponade. In Case 2, corneal epithelial debridement with EDTA was performed prior to PPV to restore adequate visualization in the setting of band keratopathy.

The decision to proceed with PPV without additional prophylactic antitumor measures (e.g., intraoperative cryotherapy to port sites or anterior chamber paracentesis for subconjunctival anti-VEGF) was based on confirmed tumor inactivity (as defined in the Tumor Inactivity Criteria subsection) and adherence to standard PPV port-site precautions (triamcinolone wound marking, wound-assisted cannula removal, and conjunctival closure).

In eyes with a history of external beam radiotherapy (EBRT), the posterior hyaloid was frequently found to be thickened and abnormally adherent to the inner retinal surface at the time of surgery, consistent with radiation-induced fibrosis of the vitreoretinal interface. In these cases, triamcinolone acetonide staining was indispensable for delineating the posterior cortical vitreous and guiding safe dissection. Extra care was taken during posterior hyaloid peeling to avoid inadvertent retinal trauma, and the threshold for using end-gripping forceps over suction-assisted separation was lower in irradiated eyes. These intraoperative findings underscore the importance of preoperative awareness of prior EBRT when planning vitreoretinal surgery in RB survivors.

### Definition of tumor inactivity and pre-surgical oncologic assessment

Tumor inactivity was defined by all of the following criteria, evaluated by the dedicated ocular oncology team at KKESH&RC (S.A.; A.K.) in the context of serial examinations under anaesthesia (EUA) performed according to our institutional surveillance protocol: A documented quiescent interval of ≥ 3 years from the last tumor treatment; absence of white fluffy tumor mass, viable vitreous seeds, or subretinal seeds on wide-field fundoscopy (Optos) and clinical EUA examination; presence of complete or near-complete tumor regression (Shields Types 1–4) with stable or decreasing lesion height and intralesional calcification on B-scan ultrasonography; absence of new subretinal fluid attributable to tumor activity; and no systemic evidence of metastatic disease on contemporaneous pediatric oncology review. Intraocular surgery was only planned after formal written documentation of confirmed tumor quiescence by the ocular oncology team. Eyes failing any of these criteria were managed with scleral buckling rather than intraocular surgery [9].

### Outcomes

The primary outcome was anatomical retinal reattachment 12 months after surgical intervention. Secondary outcomes included BCVA, patient survival, absence of postoperative tumor seeding or reactivation, and the need for enucleation after RD repair.

### Statistical analysis

Given the rarity of non-exudative RD in patients with RB and the small sample size, the statistical analysis was descriptive. Categorical variables are summarized as frequencies and percentages, and continuous variables are reported as means with ranges or standard deviations, as appropriate.

No inferential statistical tests were performed. Missing data were not imputed, and analyses were conducted using only the available records. Data were collected using a standardized Microsoft Excel (Microsoft Corp., Redmond, WA, USA) datasheet and analyzed using Stata v. 17 (StataCorp, College Station, TX, USA).

### Ethical approval

The study was approved by the Institutional Review Board of KKESH & RC (RP 25022-R). Given the retrospective design of this study, the requirement for informed consent was waived. All data were anonymized prior to analysis.

### Reporting Standards

This study was conducted in accordance with established recommendations for retrospective case series studies.

## Results

### Patient characteristics and RB background

A total of 766 patients diagnosed with RB were treated at KKESH&RC. Of these, seven eyes (0.9%) developed non-exudative RD, including RRD, TRD, and CTRRD. The cohort exhibited a slight female predominance (57%), and bilateral RB was observed in five patients (71%). The mean age at RB diagnosis was 3 years (range, 1–6 years), whereas the mean age at RD diagnosis was 21 years (range, 5–66 years). According to the ICRB, two eyes (29%) were classified as Group C, three (43%) as Group D, and one (14%) as Group E. Classification data were unavailable for one patient who received treatment at another facility. Among patients with bilateral RB, three (60%) were functionally monocular at the time of RD diagnosis owing to prior enucleation or phthisis bulbi of the fellow eye. Detailed demographic data, RB characteristics, regression patterns, ocular comorbidities, and pre-RD interventions are shown in Table 1.

**Table 1:** Baseline demographics, RB characteristics, and Pre-RD ocular status

Case	Age RB Dx (yrs)	Age RD Dx (yrs)	Sex	RB Laterality	ICRB Class	Fellow Eye	RB Treatments	RB Regression	Ocular Comorbidities Before RD	Procedures Before RD	Last RB Activity (yrs)	Cataract Sx Before RD (yrs)
1	N/A	66	M	Bilateral	C	Pthisis bulbi	N/A	Type 2 & 4	Central corneal scar; Cataract; Myopia; FTMH	None	N/A	No
**2**	1	23	F	Bilateral	D	Enucleated	SC; EBRT; PB; CRYO	Type 1	Band keratopathy; Iritis; PCO; Radiation retinopathy + keratopathy; SOAG;	PRP; Lens aspiration + IOL; AC tap	20	Yes (17)
**3**	3	22	F	Bilateral	C	Enucleated	SC; EBRT; CRYO; TTT	N/A	Myopia	Lens aspiration + IOL	8	Yes (13)
**4**	6	17	M	Bilateral	E	Regressed	SC; EBRT	Type 1	PCO; Radiation retinopathy	Lens aspiration + IOL	.	Yes (6)
**5**	3	6	F	Unilateral	D	Normal	SC; EBRT; CRYO	Type 1	Radiation + exposure keratopathy; Lagophthalmos; Myopia; Dry retinal fold; Radiation retinopathy; Cataract; Amblyopia	None	3	No
**6**	5	6	M	Unilateral	C	Normal	SC; IAC; IViC; CRYO; TTT	No regression	Amblyopia	None	0	No
**7**	3	5	F	Bilateral	D	Regressed	SC; IAC; IViC; CRYO; TTT	No regression	None	None	0	No

Abbreviations: RB = retinoblastoma; RD = retinal detachment; IIRC = International Intraocular Retinoblastoma Classification; SC = systemic chemotherapy; EBRT = external beam radiotherapy; PB = plaque brachytherapy; CRYO = cryotherapy; TTT = transpupillary thermotherapy; IAC = intra-arterial chemotherapy; IViC = intravitreal chemotherapy; PCO = posterior capsule opacification; PRP = panretinal photocoagulation; CED = corneal epithelial defect; TRD = tractional retinal detachment; ERM = epiretinal membrane; FTMH = full thickness macular hole; SOAG = secondary open angle glaucoma; N/A = Not available

### RB treatment history and tumor activity at RD diagnosis

Treatment modalities for RB vary according to the severity of the disease and the treatment era. Among patients with available records, all received systemic chemotherapy, and adjunctive therapies included cryotherapy (CRYO; 83%), external beam radiotherapy (EBRT; 67%), intra-arterial chemotherapy (IAC; 33%), intravitreal chemotherapy (IViC; 33%), plaque brachytherapy (PB; 17%), and transpupillary thermotherapy (TTT; 17%).

In two eyes (cases 6 and 7), RD developed in the context of active RB without a quiescent interval between RB and RD. In contrast, the remaining five eyes (71%) developed RD following tumor inactivity, with a complete regression period ranging from 3 to 20 years.

RRD occurred in pseudophakic eyes (*n* = 3) years after cataract surgery (range: 13–17 years); however, causality could not be inferred.

### Retinal detachment characteristics

In this study, six eyes (86%) presented with a rhegmatogenous component: four with isolated RRD and two with CTRRD. One eye (14%) exhibited isolated TRD and was managed conservatively due to longstanding macular detachment and the absence of light perception. In all cases, the macula was detached at the time of presentation. The extent of RD was confined to two quadrants in five eyes (71%), whereas total or near-total detachment was observed in two cases. Retinal breaks were predominantly located inferiorly (50%) or adjacent to regressed tumor scars (50%). In one instance of RRD (17%), no breaks were identified despite meticulous intraoperative evaluation.

PVR was identified in three of the six surgically managed eyes (50%); the grades ranged from B to CA6, as detailed in Table 2. During the follow-up period, there were no cases of postoperative PVR progression or new PVR development. During PPV, all eyes displayed adherent posterior hyaloid and dense vitreoretinal adhesions, highlighting a complex pathological vitreoretinal interface following RB. The detailed characteristics of the individual RD cases, surgical approaches, and outcomes are presented in Table 2.

**Table 2:** RD characteristics, surgical management, and clinical outcomes

Case	Type of RD	Break Location	RD Extent (Quadrants)	Macula Status	No. VR Surgeries	RD Management	PVR Grade	Seeding/Reactivation	Postoperative Complications	1-year Outcome	Baseline BCVA	Final BCVA
1	CTRRD	Inferior (prior scar)	4	Off	2	Phaco + IOL; PPV + SO; SOR; ERM peel	No PVR	No	ERM; OHT	Attached	0.9	0.9
2	RRD	Temporal (prior scar)	2	Off	4	EDTA; PPV + SO; ERM peel; SOR + steroid	CA6	No	CED; LSCD; fibrous ingrowth	Attached	0.8	0.7
3	RRD	Inferior & superior	2	Off	2	PPV + SO; SOR	B	No	Aphakia; ERM; CME; SO emulsification	Attached	0.2	0.2
4	TRD	.	2	Off	0	Observation	No PVR	.	CED; OHT; schisis; PCO	.	2.4	3.0
5	CTRRD	Inferior	2	Off	1	PPV + retinectomy + PRP + SO	CA2	No	N/A	Attached	1.4	1.4
6	RRD	Nasal (prior scar)	4	Off	1	SB + cryo	No PVR	No	Severe exposure; keratopathy	Attached	1.0	0.5
7	RRD	Not identified	2	Off	1	SB	No PVR	No	Persistent RD; active RB	Enucleated	2.3	3.0

Abbreviations: RD = retinal detachment; CTRRD = combined tractional and rhegmatogenous retinal detachment; RRD = rhegmatogenous retinal detachment; TRD = tractional retinal detachment; VR = vitreoretinal; PPV = pars plana vitrectomy; SO = silicone oil; SOR = silicone oil removal; SB = scleral buckle; ERM = epiretinal membrane; OHT = ocular hypertension; CED = corneal epithelial defect; LSCD = limbal stem cell deficiency; CME = cystoid macular edema; PRP = panretinal photocoagulation; BCVA = best-corrected visual acuity; PVR = Proliferative Vitreoretinopathy; CA = anterior circumferential proliferative vitreoretinopathy, numeral denotes clock-hours of involvement; B = grade B posterior PVR with inner retinal surface wrinkling

### Surgical management and anatomical outcomes

In this study, six of the seven eyes (86%) underwent vitreoretinal surgery. The average number of surgeries performed per eye was 1.8. PPV with silicone oil tamponade was the predominant method employed (67%), particularly in cases of CTRRD or late-onset RRD following quiescent tumors. Heavy silicone oil was used in both CTRRD cases. Nonsurgical scleral buckling was performed in two eyes with early RRD during active RB (cases 6 and 7). One eye underwent segmental scleral buckling with cryopexy, whereas the other eye underwent radial sponge placement without cryotherapy. These techniques were chosen to avoid intraocular entry during the active stage of the tumor.

At the 1-year follow-up, anatomical retinal reattachment, the primary outcome, was achieved in five of the six operated eyes (83.3%). One eye, which had active RB at the time of RD diagnosis, progressed despite scleral buckling and was enucleated 1 month later due to insufficient tumor control. No instances of RB seeding or tumor reactivation were observed after intraocular surgery.

Among the eyes that underwent PPV, the quiescent interval before RD ranged from 3 to 20 years (cases 2, 3, and 5; case 1 had an unquantifiable interval due to incomplete prior records). The interval between RD diagnosis and PPV ranged from 3 to 10 days. Therefore, the total interval from the last documented RB activity to PPV exceeded 3 years in all cases with available data, well above the minimum threshold of 1–2 years recommended in the literature [3].

### Postoperative complications

**Fig. 1:**
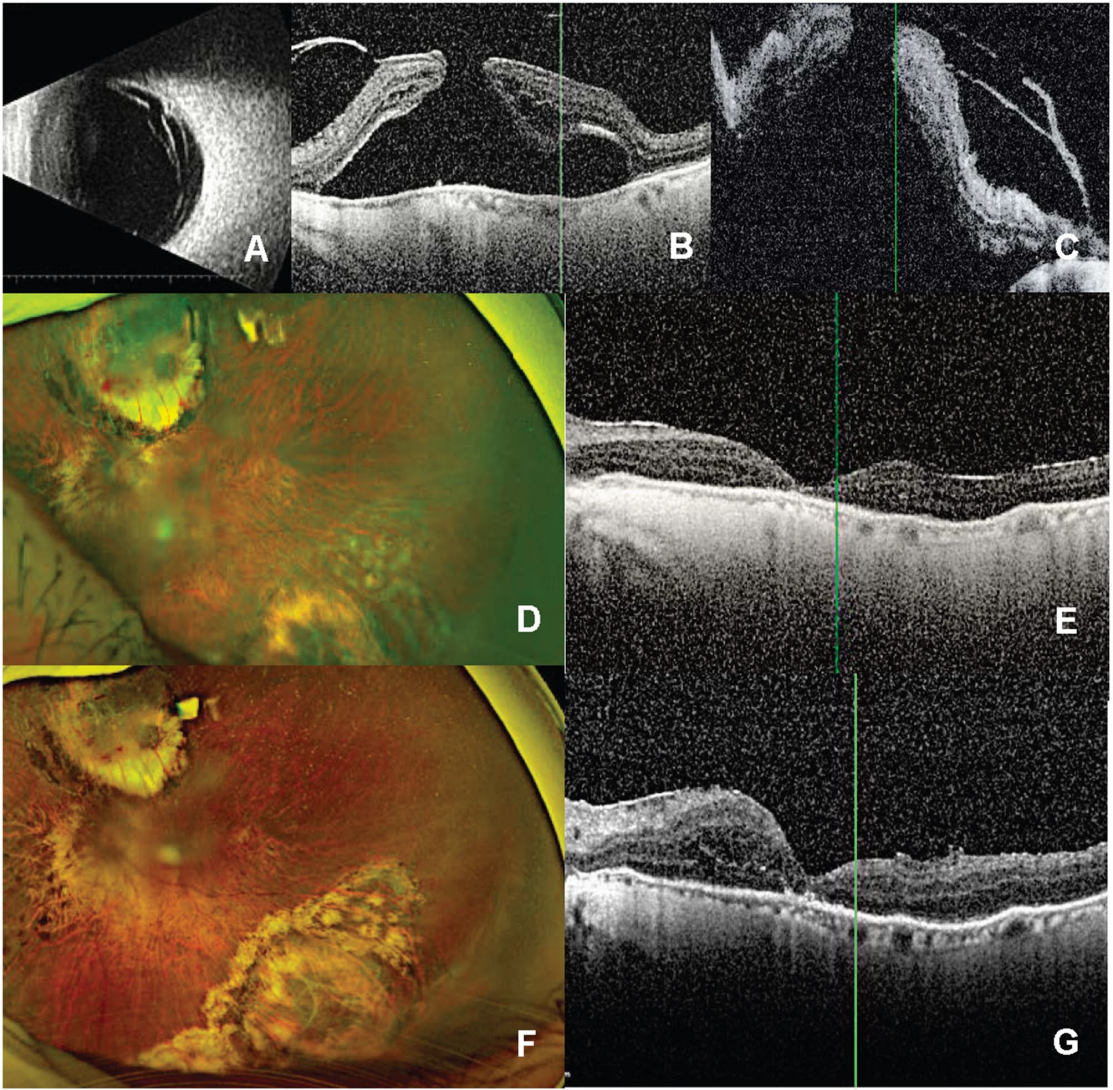
Case 1. (**A**) A B-scan ultrasound image obtained several years before the development of RD reveals the presence of tractional membranes. (**B**) An optical coherence tomography (OCT) scan conducted prior to the development of RD revealed the presence of an epiretinal membrane (ERM), which induced progressive foveal traction and a full-thickness macular hole (FTMH). (**C**) OCT at the time of RD diagnosis showing a macula-off CTRRD with an ERM and FTMH. (**D**) An Optos fundus photograph on the first postoperative visit showing a flat retina under silicone oil. (**E**) OCT on the first postoperative visit showing a flat macula under oil and a closed FTMH. (**F**) An Optos fundus photograph taken during the first postoperative visit following oil removal and epiretinal membrane (ERM peeling revealed a flat retina. (**G**) OCT conducted during the initial postoperative visit following oil removal and ERM peeling revealed a flat macula accompanied by parafoveal macular edema

Postoperative complications were observed in five (83%) of the six eyes. The most prevalent complication was a persistent corneal epithelial defect (CED), which occurred in 50% of the cases, followed by epiretinal membrane formation (33%). Two eyes with persistent CED had a pre-existing severe deficiency of limbal stem cells that progressed to corneal vascularization and scarring. One monocular patient ultimately required the implantation of a Boston keratoprosthesis in conjunction with silicone oil removal and membrane peeling, resulting in anatomical stabilization but limited visual recovery (Fig. 1). Additional complications included silicone oil emulsification, cystoid macular edema, and ocular hypertension (each at 17%), all of which were successfully managed. A comprehensive overview of postoperative complications and management strategies is presented in Table 2.

### Visual outcomes and follow-up

The mean BCVA decreased from 0.98 logMAR before RD to 1.62 logMAR at the time of diagnosis. After treatment, BCVA returned to baseline or improved in five eyes (71%). Poor visual outcomes were correlated with active RB at the time of RD diagnosis, tractional RD, advanced corneal disease, and suboptimal initial vision. All the patients were alive at the last follow-up. The duration of postsurgical surveillance varied by case (Table 2). Among the eyes that underwent PPV, the follow-up after the first PPV ranged from 7 to 10 years (mean 8 years). No evidence of tumor seeding or reactivation was detected in any eye during the surveillance period. The two eyes treated with scleral buckling were followed for 4 and 3 years, respectively (the latter was enucleated at 1 month due to tumor progression). Histopathological analysis of the enucleated eye revealed choroidal invasion and high-risk features without evidence of extrascleral extension.

### Integration of imaging findings

**Fig. 2:**
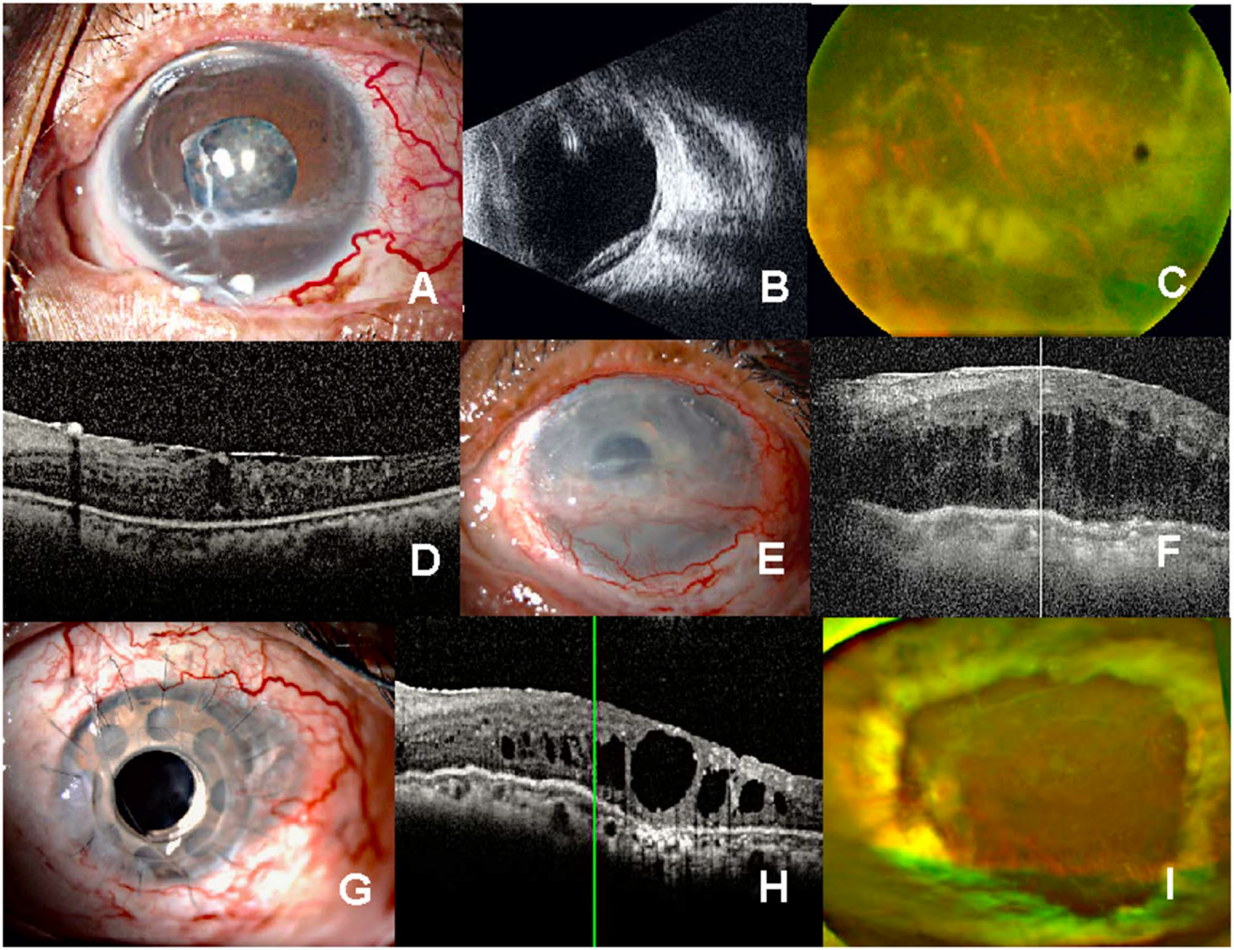
Case 2. (**A**) Slit-lamp photograph showing a preoperative corneal scar and posterior chamber intraocular lens with posterior capsular opacity. (**B**) B-scan ultrasound performed at the time of RD diagnosis. (**C**) Optos fundus photography performed during the initial postoperative visit revealed a flattened retina beneath the oil. (**D**) During the initial postoperative visit, OCT revealed a flat macula beneath the oil, with cystic changes. (**E**) A slit-lamp photograph was taken two years postoperatively, demonstrating progression in corneal scarring and vascularization. (**F**) OCT performed two years postoperatively revealed a flat macula and an ERM accompanied by macular edema. (**G**) Slit-lamp photograph taken during the initial postoperative visit following combined anterior segment and vitreoretinal surgery, which included Boston keratoprosthesis, silicone oil removal, epiretinal membrane peeling, and triamcinolone injection. (**H**) Four months after combined anterior segment and vitreoretinal surgery, OCT revealed a flat macula with reduced macular edema. (**I**) An Optos fundus photograph was taken ten months following combined anterior segment and vitreoretinal surgery, demonstrating a flattened retina

**Fig. 3:**
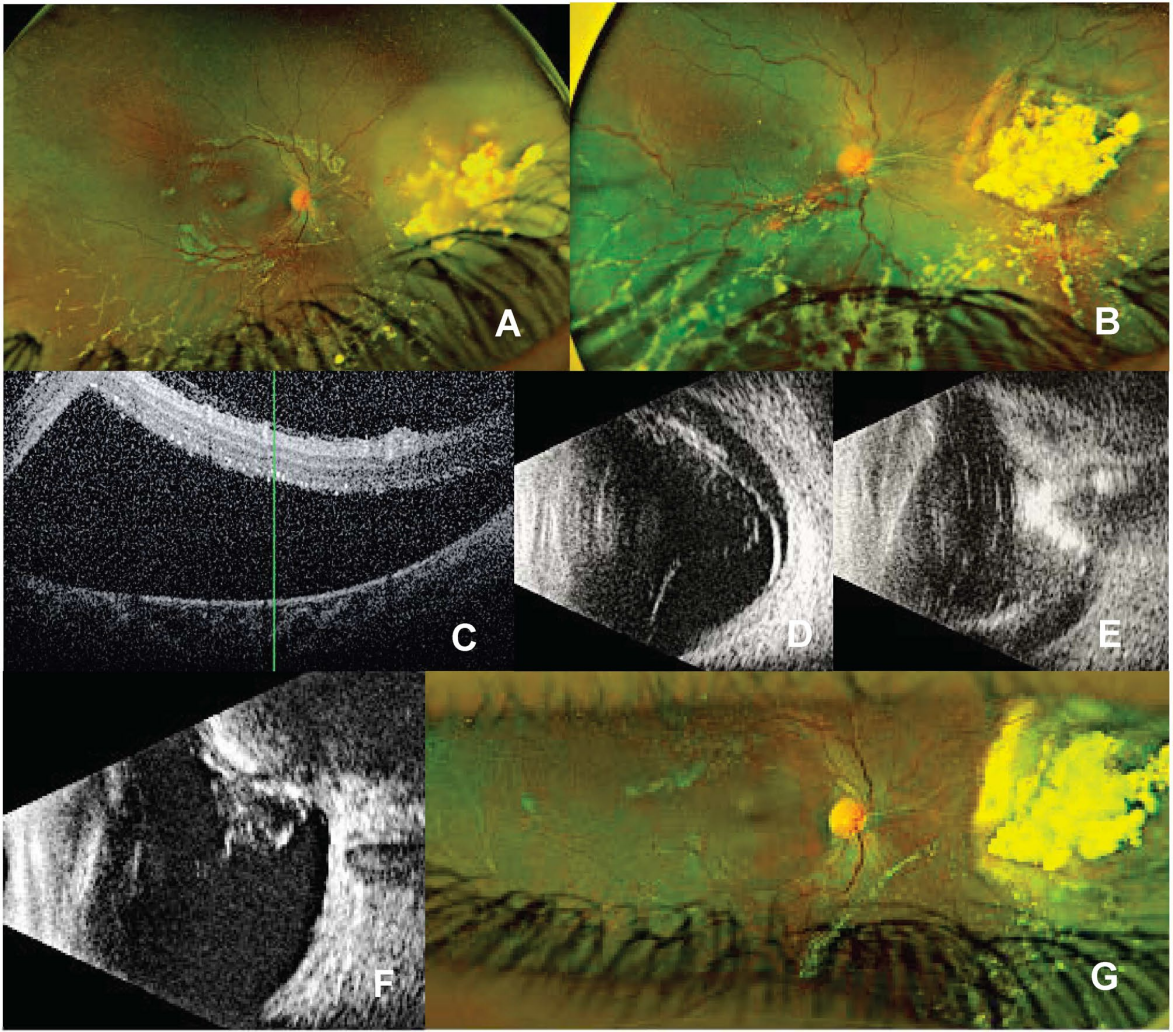
Case 6. (**A**) Optos fundus photograph taken prior to the diagnosis of RD reveals an active RB lesion accompanied by vitreous seeds and localized exudative RD. (**B**) and (**C**) Two months after cryotherapy, Optos fundus photographs and OCT revealed a regressing RB lesion and progressive retinal detachment (macula off), indicating a diagnosis of RRD. (**D**), (**E**) B-scan ultrasound showing a RB lesion with associated RD. (**F**) One month postoperatively, B-scan ultrasound revealed a regressing RB lesion, segmental buckle indentation, and a flat retina. (**G**) Optos fundus photograph taken two years postoperatively revealed a flat retina and inactive RB

Figures 1, 2 and 3 show multimodal imaging findings. Figure 1 focuses on Case 2, highlighting complex anterior segment morbidity following RD repair and the integration of the keratoprosthesis with vitreoretinal surgery. Figure 2 illustrates the progression of chronic TRD in Case 1, emphasizing the development of progressive vitreoretinal traction, formation of a full-thickness macular hole, and subsequent postoperative anatomical restoration. Figure 3 depicts the early development of RRD in the context of active RB in Case 6, demonstrating the feasibility of scleral buckling, followed by tumor regression and stable, long-term retinal stability.

No instances of RB seeding or tumor reactivation were observed after intraocular surgery within the available follow-up period; however, the small number of cases limits the statistical power to detect rare events, and continued long-term oncologic surveillance remains essential.

## Discussion

Vitreoretinal complications after RB treatment are well documented and arise from a complex interplay between tumor regression, globe-salvaging therapies, and long-term treatment-induced alterations in the retina and vitreous humor [3]. Previous studies have reported vitreoretinal complications in up to 6–11% of conservatively treated eyes with RB, including retinal tears, RRD, TRD, CTRRD, vitreoretinal traction bands, and subretinal fibrosis [1, 3]. In contrast, the overall prevalence of non-exudative RD in our cohort was 0.9%, comprising 0.8% RRD and 0.4% TRD, which is lower than that reported in most published series [11–14].

This discrepancy likely reflects regional variations in the presentation and management of RB. In Saudi Arabia and similar regions, RB has traditionally been diagnosed at a more advanced stage, often necessitating primary enucleation rather than globe-sparing therapy, thereby reducing the number of eyes at risk of long-term vitreoretinal sequelae [15, 16]. With increasing awareness, earlier diagnosis, and broader adoption of conservative treatments, the prevalence of secondary vitreoretinal complications is expected to increase [16]. Additionally, extramacular or clinically silent TRDs may remain underdiagnosed, contributing to the variability in the prevalence reported across studies [5, 6]. However, this remains speculative and requires prospective epidemiological data for confirmation.

A significant finding of our study was the notably delayed onset of RD. This mean age at RD diagnosis was 21 years, which is considerably older than that reported in most previous studies, where RD typically manifests during childhood or early adolescence, even in cases described as late presentations [17–24]. This observation contributes to the existing body of knowledge by illustrating that eyes with regressed RB remain susceptible to secondary RD several decades after initial tumor control, emphasizing the necessity of lifelong surveillance in globe-salvaged RB survivors.

In alignment with previous studies, all patients in our cohort presented with advanced RB (ICRB groups C–E), corroborating the link between an elevated tumor stage and an increased risk of non-exudative RD [21, 25]. Advanced stages of the disease are generally treated with multimodal therapy, which predisposes patients to cumulative retinal damage and pathological changes at the vitreoretinal interface. In our series, eyes classified as group C exhibited more favorable anatomical and functional outcomes than those in groups D and E, consistent with earlier reports of poorer outcomes in more advanced stages [2–4, 11]. The pattern of tumor regression also appears to affect the risk of developing RD. Shields et al. identified four regression patterns, with type 1 (calcified remnant) most frequently associated with secondary RRD [26]. Most late RD cases in our cohort exhibited type 1 regression. We hypothesize that calcified tumor edges, together with treatment-induced retinal thinning, may predispose patients to atrophic breaks; however, this association cannot be confirmed in the present series and requires prospective evaluation.

Globe preservation therapies are integral to the pathophysiology of RRD. Cryotherapy, especially when combined with systemic or local chemotherapy, is a well-documented risk factor, with retinal breaks frequently occurring near cryotherapy scars [2, 6]. Rapid tumor regression following intra-arterial or intravitreal chemotherapy may further predispose individuals to breakage at focal treatment sites [25]. Radiation therapy contributes to this risk through mechanisms such as vascular occlusion, ischemia, progressive radiation retinopathy, and fibrovascular proliferation, which may ultimately result in TRD [27]. In our cohort, all patients with late RD underwent external beam radiation, with most exhibiting radiation retinopathy. Cataract extraction, often performed for radiation-induced cataracts, is an additional risk factor for late-onset RRD [28]. Two of our late RRD cases developed RD more than a decade after cataract surgery, which is consistent with the long-term risks described in the literature.

The timing of RD relative to tumor activity is a key determinant of both the complexity and management strategy. Early RRDs occurring in active RBs were morphologically simpler and lacked tractional components, whereas late RDs following tumor regression were uniformly complex, often involving TRD or an abnormal vitreoretinal interface. Even in late RRDs without overt traction, markedly adherent posterior hyaloid and pathological vitreous attachments have been consistently noted intraoperatively, increasing surgical complexity, as previously reported [1, 8].

PVR was identified in three of the six surgically managed eyes (50%) in this series, with grades ranging from B to CA6 [10]. This proportion exceeds the 5–10% incidence in the general RRD population [10], consistent with the propensity for fibroproliferative responses in RB-treated eyes. PVR after RB treatment likely results from the combined effects of cryotherapy, radiotherapy, and intraocular inflammation at the vitreoretinal interface [7]. The anterior PVR grades in Cases 2 and 5 (CA6 and CA2) reflect predominantly anterior proliferation, consistent with vitreous base involvement after extensive focal treatment. In RB-treated eyes, early proliferation can be challenging to differentiate from recurrent tumors, necessitating preoperative oncologic clearance [12]. Despite the higher prevalence of PVR, anatomical reattachment was successfully achieved in all three affected eyes after one year. This outcome suggests that satisfactory results can be attained with proper surgical planning, including the use of silicone oil tamponade and relaxing retinotomy. Owing to the small sample size, it is not possible to draw any conclusions about the natural history or progression risk of PVR in this oncologic context.

Surgical management should be tailored to individual patients. In cases of early RRD associated with active RB, scleral buckling is the preferred method because it avoids intraocular penetration and reduces the risk of tumor dissemination. Nondrainage techniques are recommended, and the use of segmental or radial elements may mitigate acute intraocular pressure (IOP) elevation compared to encircling bands [7]. When encircling bands are necessary, anterior chamber paracentesis should be avoided in eyes with viable tumor cells. Instead, preoperative osmotic agents and postoperative carbonic anhydrase inhibitors are safer alternatives for managing IOP [8].

PPV remains a subject of debate because of historical accounts of tumor seeding, particularly in cases involving eyes with undiagnosed active RB [29]. Therefore, intraocular surgery is contraindicated in the presence of active disease and should only be considered following a documented quiescent period of 1–2 years [3]. In our cohort, all instances of late RDs occurred in eyes with inactive RB and were effectively managed using small-gauge PPV and silicone oil tamponade, resulting in high anatomical success rates. Triamcinolone vitreous staining is routinely employed to enhance the visualization of abnormal patterns of vitreoretinal adhesions. The posterior cortical vitreous was detached from the retina to the greatest extent. Extended peripheral shaving was performed under scleral depression. Silicone oil was preferred over gas tamponade due to the patient’s monocular status and potential risk of recurrence. Heavy silicone oil is specifically used for cases involving inferior breaks [30].

Radiation-induced changes to the posterior vitreous represent a recognized surgical challenge in eyes with irradiated RB. EBRT, which was used in four of our seven cases, is known to cause progressive thickening and fibrocellular transformation of the vitreous cortex, resulting in firm vitreoretinal adhesions that increase the difficulty and risk of posterior hyaloid separation during PPV [8, 13]. In our EBRT-treated cases, the posterior hyaloid was notably thickened and required prolonged triamcinolone-guided dissection. These observations should prompt surgeons to anticipate increased intraoperative complexity in irradiated eyes and to routinely employ staining-assisted vitrectomy agents to facilitate safe posterior vitreous removal.

Although the anatomical outcomes were favorable, postoperative morbidity was prevalent, attributable to both the aggressive nature of prior therapies and the complexity of the surgical procedures. Radiation keratopathy emerged as the most common complication, occasionally requiring keratoprosthesis implantation, which is consistent with previous studies [27, 31]. The frequent occurrence of epiretinal membrane formation highlights the highly proliferative environment in post-RB eyes and underscores the necessity of diligent postoperative optical coherence tomography monitoring [1, 8].

In all eyes undergoing PPV, tumor quiescence was formally certified by the Ocular Oncology Team using a structured preoperative assessment that incorporated clinical EUA findings, wide-field fundoscopy, B-scan ultrasonography, regression pattern documentation, and the absence of viable seeds, with a minimum quiescent interval of 3 years. Given this rigorous preoperative oncologic evaluation, we did not implement intraoperative prophylactic measures (cryotherapy or melphalan irrigation), as the available evidence does not support their routine use in confirmed inactive eyes, and melphalan irrigation carries its own risk of retinal toxicity [17]. However, we emphasize that this approach is only justifiable when quiescence has been confirmed by an experienced Ocular Oncologist, and that any uncertainty regarding tumor activity remains an absolute contraindication to intraocular surgery.

Notably, no instances of tumor seeding or reactivation were observed in this small series following vitreoretinal surgery, which is consistent with, but does not by itself establish, the oncologic safety of scleral buckling and PPV when these procedures are appropriately timed and selected [3, 6, 28]. These findings add to the growing body of observational evidence but must be interpreted within the constraints of a small retrospective case series with limited statistical power to detect rare events. However, continued vigilance is imperative, as rare cases of delayed reactivation have been reported [29].

### Limitations and clinical implications

This descriptive retrospective case series was not intended to determine the incidence or prevalence of non-exudative RD in globe-salvaged RB eyes. The figure of 766 institutional RB patients highlights the scarcity of the condition; however, it is not a formal denominator, which would require a dedicated cohort study. The small sample size (*n* = 7 eyes) underscores the rarity of this complication rather than inadequate case detection; however, it limits statistical analysis and generalizability, further impacted by variability in prior treatments, RD types, and ocular comorbidities. The retrospective single-center design risks information bias, although the national referral pattern improves representativeness.

No tumor seeding or reactivation was observed, offering reassurance; however, this should not be interpreted as evidence for the overall safety of intraocular surgery in treated RB eyes. With only four eyes undergoing PPV after verified tumor quiescence, the study lacked statistical power for rare adverse events.

These findings contribute to, but do not confirm, oncologic safety. Larger prospective multicenter registries with standardized criteria and long-term follow-up are necessary to reach definitive conclusions.

## Conclusion

In this small retrospective series of seven eyes, anatomical retinal reattachment, the primary outcome, was achieved in 83.3% of the operated eyes at one year after surgery, with no tumor seeding or reactivation observed during the follow-up.

Non-exudative RD following RB treatment is an infrequent but potentially vision-threatening complication of globe-preserving therapy. In this small retrospective series, early RRD occurring during active disease was managed with extraocular scleral buckling, whereas late or complex RDs following confirmed tumor quiescence were addressed with PPV and intraocular tamponade. No tumor seeding or reactivation was observed in any case; this finding is encouraging but should be interpreted with caution given the limited sample size. Disease stage and cumulative treatment exposure appeared to influence both the risk of RD and surgical outcomes, a relationship that warrants further investigation in larger prospective cohorts. These observations support the need for meticulous case selection, documented tumor inactivity before any intraocular procedure, and lifelong surveillance of globe-salvaged RB survivors. Multicenter studies with extended follow-up are necessary to establish evidence-based management guidelines for this rare and challenging condition.

## Electronic Supplementary Material

Below is the link to the electronic supplementary material.

Supplementary Material 1

## Data Availability

The datasets generated and/or analyzed in the current study are available from the corresponding author upon reasonable request.
